# Predicting postoperative adhesive small bowel obstruction in infants under 3 months with intestinal malrotation: a random forest approach

**DOI:** 10.1016/j.jped.2024.11.011

**Published:** 2025-01-21

**Authors:** Pengfei Chen, Haiyi Xiong, Jian Cao, Mengying Cui, Jinfeng Hou, Zhenhua Guo

**Affiliations:** a Department of General Surgery and Neonatal Surgery, Liangjiang Wing, Children's Hospital of Chongqing Medical University, National Clinical Research Center for Child Health and Disorders, Ministry of Education Key Laboratory of Child Development and Disorders, Chongqing Key Laboratory of Pediatrics, Chongqing, China; b Department of Pediatrics, Women and Children's Hospital of Chongqing Medical University, Department of Pediatrics, Chongqing Health Center for Women and Children, Chongqing, China

**Keywords:** Adhesive small bowel obstruction, Intestinal malrotation, Machine learning, Random forest, Clinical decision-making

## Abstract

**Objective:**

This study aimed to develop a predictive model using a random forest algorithm to determine the likelihood of postoperative adhesive small bowel obstruction (ASBO) in infants under 3 months with intestinal malrotation.

**Methods:**

A machine learning model was used to predict postoperative adhesive small bowel obstruction using comprehensive clinical data extracted from 107 patients with a follow-up of at least 24 months. The Boruta algorithm was used for selecting clinical features, and nested cross-validation tuned and selected hyper-parameters for the random forest model. The model's performance was validated with 1000 bootstrap samples and assessed using receiver operating characteristic (ROC) analysis, the area under the ROC curve (AUC), sensitivity, specificity, precision, and F1 score.

**Results:**

The random forest model demonstrated high diagnostic accuracy with an AUC of 0.960. Significant predictors of ASBO included pre-operative white blood cell count (pre-WBC), mechanical ventilation (MV) duration, surgery duration, and post-operative albumin levels (post-ALB). Partial dependence plots showed non-linear relationships and threshold effects for these variables. The model achieved high sensitivity (0.805) and specificity (0.952), along with excellent precision (0.809) and a robust F1 score (0.799), indicating balanced recall and precision performance.

**Conclusion:**

This study presents a machine learning model to accurately predict postoperative ASBO in infants with intestinal malrotation. Demonstrating high accuracy and robustness, this model shows great promise for enhancing clinical decision-making and patient outcomes in pediatric surgery.

## Introduction

Intestinal malrotation is a congenital disorder characterized by abnormal embryonic midgut development, resulting in disrupted bowel rotation and fixation. This leads to anatomical abnormalities that increase the risk of complications such as volvulus and obstruction. Approximately 1 in 500 live births are affected by this condition,^1^, ^2^, ^3^ which is usually diagnosed during infancy or early childhood. The current standard treatment for intestinal malrotation is surgical intervention, aiming to correct the anatomical abnormalities and minimize the risk of complications. However, even with advancements in surgical techniques and perioperative care, some patients may develop postoperative complications, including adhesive small bowel obstruction (ASBO).

ASBO frequently occurs following abdominal surgery),^4^, ^5^, ^6^ such as that for intestinal malrotation. It is caused by fibrous bands, known as adhesions, forming between abdominal organs and tissues. These adhesions can lead to intestinal obstruction through compression or torsion of the bowel, producing symptoms like abdominal pain, distension, and emesis. The incidence of ASBO post-surgery for intestinal malrotation ranges from 8 % to 29 %.^7^, ^8^, ^9^, ^10^, ^11^, ^12^

Diagnosing ASBO currently relies heavily on clinical judgment and imaging techniques).^13^^,^^14^ However, these methods have limitations in specificity and can be challenging due to the subtle presenting symptoms in infants. Consequently, there is growing interest in using machine learning algorithms to enhance diagnostic accuracy and support decision-making in the early identification of ASBO.

Machine learning models, which have become increasingly popular in various fields, offer advantages over traditional statistical analysis methods. They can analyze nonlinear relationships between data, proving beneficial in disease diagnosis, subtype identification, and biomarker discovery).^15^, ^16^, ^17^ Random forest is a machine learning algorithm that uses an ensemble of decision trees to make predictions. Each decision tree in the “forest” works independently, analyzing different parts of the data to classify outcomes or make predictions. The final result is determined by combining the outputs of all the trees.

This study aims to apply a random forest algorithm to develop a predictive model for early identification of ASBO in infants under three months who have undergone surgery for intestinal malrotation. By analyzing a range of clinical parameters, the goal is to establish a model that effectively predicts the likelihood of ASBO. This will aid in timely clinical decision-making, optimize patient management, and ultimately improve outcomes for this vulnerable patient group.

## Material and methods

**Figure 1:**
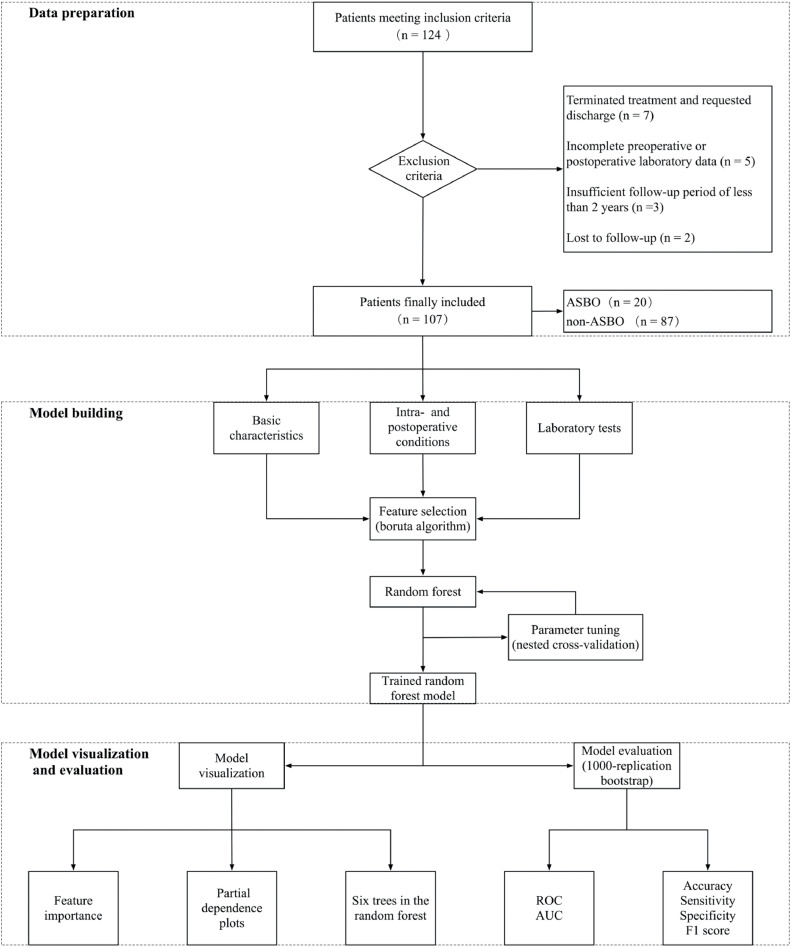
Architecture of the framework of this study.

This study's framework, depicted in Figure 1, includes three main parts: data preparation, model building, and model visualization and evaluation.

### Patient selection

Patients treated at the Children's Hospital of Chongqing Medical University from January 2012 to December 2020 were enrolled in this study. All participants were diagnosed with intestinal malrotation and had undergone surgery. They were followed up for at least two years postoperatively, with categorization based on the occurrence of ASBO. The Ethics Committee of the Children's Hospital of Chongqing Medical University approved this study (File No 57–2, 2022).

### Inclusion and exclusion criteria

The study included patients aged under three months, definitively diagnosed with intestinal malrotation, and who had undergone Ladd's procedure at the hospital. Exclusion criteria encompassed patients with incomplete clinical data, those who discontinued treatment or left the hospital voluntarily, and those with less than two years of follow-up.

### Definition of ASBO

Adhesive small bowel obstruction (ASBO) is characterized by symptoms such as vomiting, abdominal pain, and distension. Its diagnosis is confirmed by abdominal X-rays showing significant intestinal loop dilation and air-fluid levels. ASBO commonly results from fibrous adhesions in the small intestine, often occurring after abdominal surgeries.

### Predictor variables

The present study carefully selected a wide range of factors for a comprehensive analysis that includes both clinical observations and laboratory data. Factors considered include the rotation angle observed during surgery, and demographic and physiological data like gender, age in days, mode of delivery, birth weight, and admission weight. Surgical evaluation focused on the duration of the procedure, while postoperative care included mechanical ventilation duration (MV duration). Laboratory analysis, covering both preoperative and postoperative periods, involved parameters such as white blood cell count (WBC), neutrophil-to-lymphocyte ratio (NLR), red blood cell count (RBC), hemoglobin (HB), platelet count (PLT), C-reactive protein (CRP), total bilirubin (TBIL), and blood urea nitrogen (BUN), with postoperative values collected between 5 and 7 days after surgery. The liver function test enzyme index (LFTEI) was calculated from alanine aminotransferase (ALT) and aspartate aminotransferase (AST) levels and the time to start oral feeding (SOF) was recorded as an indicator of postoperative recovery.

### Feature selection

In this study, the authors used the Boruta algorithm for feature selection to identify significant predictors of ASBO in patients with intestinal malrotation. Designed for high-dimensional datasets, the Boruta algorithm creates shadow features by generating random copies of the original features. It then compares the importance of each real feature to these shadow features using a random forest classifier. Features less important than the most significant shadow feature are iteratively removed, ensuring that only those with statistically significant contributions to the model's predictive power are retained.

### Parameter tuning

After feature selection, the authors utilized the random forest algorithm for modeling, to optimize the random forest model, we applied a nested cross-validation approach combined with grid search. This approach addresses the challenges posed by the limited sample size. Instead of partitioning the dataset into distinct training and testing sets, the authors implemented 4-fold cross-validation in both the inner and outer loops of the nested procedure. This approach provides a robust estimate of model performance by evaluating various parameter combinations across different data subsets.

In the inner loop, grid search systematically explored parameter settings, with each configuration evaluated through 4-fold cross-validation. The results were visualized using heatmaps, facilitating the identification of optimal parameter combinations based on performance metrics. This method minimizes the risk of overfitting by ensuring the selected parameters generalize effectively across the entire dataset, thereby enhancing predictive performance.

### Model visualization and evaluation

The authors employed feature importance metrics and partial dependence plots to visualize and interpret the model. Feature importance metrics identified variables significantly influencing predictions, while partial dependence plots illustrated the effects of key features on the model's output. To further enhance interpretability, the authors visualized six individual trees from the random forest model, providing insights into the contributions of different trees to the final predictions.

For model evaluation, the authors applied a bootstrap method with 1000 replications, yielding reliable estimates of accuracy, sensitivity, specificity, F1 score, and the area under the ROC curve (AUC). The ROC curve analysis assessed the model's ability to discriminate between classes, serving as a robust diagnostic tool. Together, these visualization and evaluation techniques ensured the robustness and reliability of the model.

### Statistical analysis and software tools

Continuous variables were presented as mean ± standard deviation (SD) or median (p25, p75), depending on their distribution. Categorical variables were expressed as numbers and percentages. The authors used the *t*-test or Wilcoxon rank-sum test for continuous variables, and the chi-square test for categorical variables, based on data distribution.

Descriptive statistics and data management were performed using IBM SPSS Statistics (version 27). Feature selection with the Boruta algorithm was conducted in R using the ‘Boruta’ package (version 4.3.1). Random forest modeling, visualization, and evaluation were carried out in Python (version 3.11).

## Results

In this study, 107 infants were included. Table 1 presents the demographic and clinical characteristics of both the non-ASBO (*n* = 87) and ASBO (*n* = 20) groups. Notably, there is a female majority in both groups, with a higher percentage of females in the ASBO group. A significant finding is the longer duration of surgery in the ASBO group compared to the non-ASBO group. Correspondingly, the ASBO group required extended MV duration postoperatively. Hematological analysis revealed significant changes in specific blood parameters when comparing pre-and post-operative values in both groups. Particularly, the ASBO group exhibited a substantial postoperative increase in WBC, potentially indicating a stronger inflammatory or stress response to surgery. Additionally, the NLR, another critical systemic inflammation indicator, was notably higher in the ASBO group after surgery.

**Table 1:** Demographic and clinical characteristics.

	non-ASBO, *n* = 87	ASBO, *n* = 20
gender		
male	21 (24.1 %)	2 (10 %)
female	66 (75.9 %)	18 (90 %)
mode of delivery		
vaginal delivery	28 (32.2 %)	10 (50 %)
cesarean section	59 (67.8 %)	10 (50 %)
rotation angle, degree	360 (360,540)	540 (360,675)
days of age	8 (3,17)	7.5 (5,28)
birth weight, kg	3.21 (2.90,3.50)	3.29 (3.00,3.45)
admission weight, kg	3.05 (2.70,3.60)	3.09 (2.72,3.98)
surgery duration, minutes	65 (50,80)	108 (75,139)
MV duration, hours	5.5 (3.6,11.4)	15.9 (11.6,21.6)
pre-WBC, *10^9/L	6.6 (4.9,8.9)	9.7 (7.8,14.5)
pre-NLR	1.33 (0.98,2.14)	1.92 (1.22,2.7)
pre-RBC, *10^12/L	3.7 (3.2,4.2)	3.5 (3.1,3.8)
pre-HB, g/L	122 (108,137)	116 (100,132)
pre-PLT, *10^9/L	330 (247,420)	346 (224,426)
pre-CRP, mg/L	4 (4,4)	4 (4,4)
pre-TBIL, µmol/L	127.8 (51.9,179.7)	140.9 (70.5,185.5)
pre-LFTEI, U/L	47.3 (39.6,61.1)	48.3 (40.7,65.5)
pre-ALB, g/L	28.9 (23.4,34.0)	33.0 (29.0,36.2)
pre-BUN, mmol/L	3.4 (2.4,5.1)	5.0 (3.8,7.0)
time to SOF, days	6 (4,7)	7 (5,8)
post-WBC, *10^9/L	6.5 (5.0,7.8)	10.0 (7.7,13.1)
post-NLR	1.12 (0.81,1.76)	1.16 (0.91,1.80)
post-RBC, *10^12/L	3.4 (3.1,3.7)	3.2 (3.0,3.8)
post-HB, g/L	111 (99,121)	109 (95,120)
post-PLT, *10^9/L	380 (276,480)	409 (322,450)
post-CRP, mg/L	4 (4,4)	4 (4,4)
post-TBIL, µmol/L	45.1 (15.5,113.1)	76.9 (34.6,120.2)
post-LFTEI, U/L	43.1 (36.2,56.0)	54.4 (37.4,100.3)
post-ALB, g/L	38 (35,42)	34 (32,37)
post-BUN, mmol/L	5.2 (3.6,6.5)	5.3 (3.6,7.9)

Utilizing the Boruta algorithm with parameters “maxRuns = 100”, and “*p*-Value = 0.01”, optimal features were selected for predicting the target variable. Supplementary Figure S1 displays these results: the x-axis lists the evaluated features, and the y-axis shows their importance. Shadow attributes' importance, a baseline metric for feature selection derived from the algorithm, is depicted by three blue boxplots showing their minimum, mean, and maximum values. Significant features included in the model are highlighted in green, while those excluded are shown in red. Yellow boxplots represent tentative features, with no definitive recommendation from the algorithm for their inclusion or exclusion. Of the 29 variables analyzed, 6 were identified as significant, 1 remains tentative, and 22 were excluded. The significant features are surgery duration, MV duration, pre-operative WBC, pre-operative BUN, post-operative LFTEI, and post-operative ALB, all contributing notably to the model's predictive accuracy.

Supplementary Figure S2 demonstrates the application of nested cross-validation and grid search methods for selecting the optimal hyperparameter combination for the model, which was determined to be “max_depth = 2” and “n_estimators = 80”. This combination achieved a model accuracy of 0.9004. Furthermore, the random forest model was configured with the “class_weight” set to ‘balanced’ and the “criterion” set to “entropy”, with all other parameters at their default settings.

**Figure 2:**
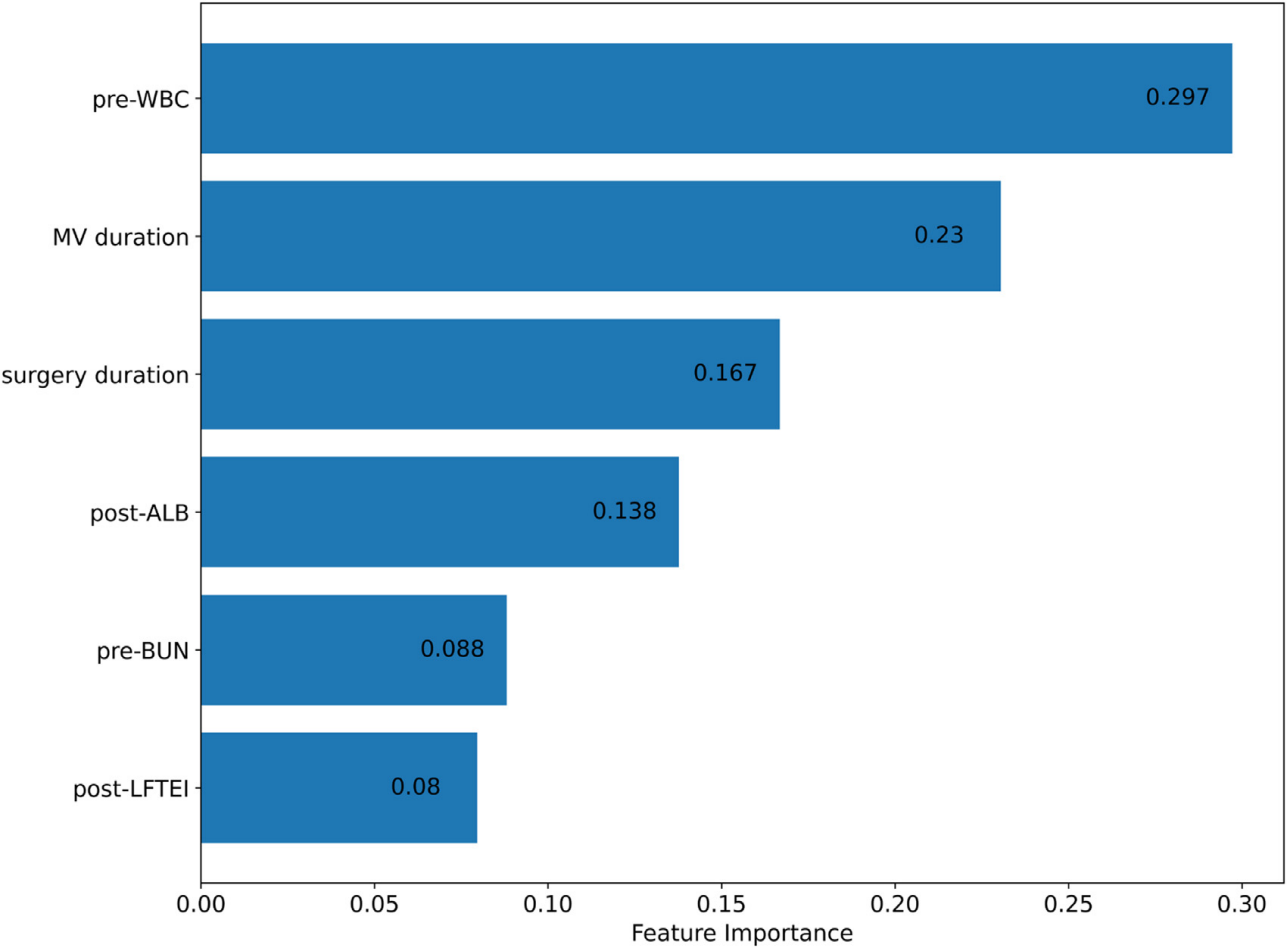
Feature importance ranking for ASBO prediction in random forest model.

After finalizing the optimal hyperparameter settings, a random forest model was constructed. The feature importance graph (Figure 2) displays the relative significance of each variable in the model. Pre-operative white blood cell count (pre-WBC) is the most influential feature with an importance score of approximately 0.297, highlighting its strong predictive power. The second most significant predictor is mechanical ventilation duration (MV duration), scoring around 0.23. Surgery duration follows with an importance score of 0.167, and post-operative albumin levels (post-ALB) also contribute notably with a score of 0.138. Pre-operative blood urea nitrogen (pre-BUN) and post-operative liver function test enzyme index (post-LFTEI) have importance scores of 0.088 and 0.08, respectively, indicating their meaningful but lesser impact.

The partial dependence plots (PDPs, Supplementary Figure S3) illustrate the relationships between predictor variables and the model's predictions. It is important to note that PDPs assume the independence of the predictor variable being analyzed from all other predictors in the model. Both surgery duration and MV duration show an increasing influence on the model's output, with MV duration plateauing after 10 h, indicating a threshold effect. Pre-WBC levels sharply increase in impact up to a certain point, suggesting a strong influence within specific ranges. Pre-BUN demonstrates a consistent, gradual influence, while post-LFTEI's impact increases up to a certain level and then stabilizes. Notably, post-ALB levels display a non-linear effect, indicating a complex interaction with the outcome, where only specific ranges significantly alter the prediction. These patterns underscore the nuanced contribution of each clinical factor to the predictive model.

The random forest model, comprising 80 decision trees, is exemplified by six trees in Supplementary Figure S4, providing insight into the model's decision-making process. These trees indicate that features such as pre-WBC, surgery duration, and MV duration are key splitting factors, signifying their substantial role in the model.

**Figure 3:**
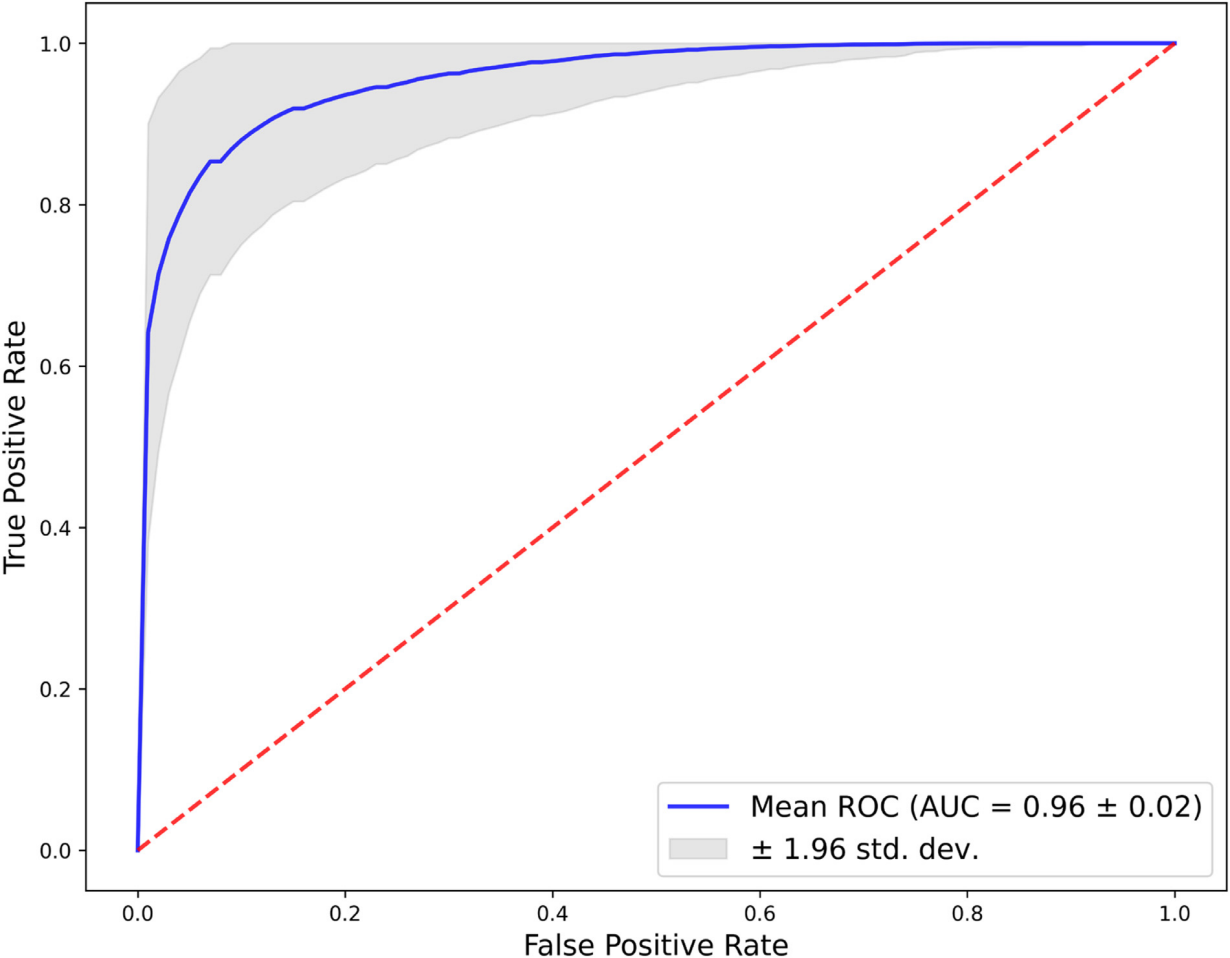
Bootstrap aggregate roc curve for ASBO prediction model performance.

The ROC curve (Figure 3), derived from 1000 bootstrap replications, assesses the model's discriminative ability. The mean ROC curve, shown by the blue line, demonstrates an excellent ability to differentiate between positive and negative classes with an AUC of 0.96 ± 0.02, indicative of outstanding model performance. The grey area around the mean ROC curve, representing the 95 % confidence interval, reflects the precision of the AUC estimation. The curve's proximity to the top left corner and its elevation above the diagonal red dashed line underscore the model's strong predictive accuracy. The narrow confidence interval highlights the stability and consistency of the model across different samples. Sensitivity, specificity, precision, and F1 score, along with their 95 % confidence intervals, are detailed in Supplementary Table S1.

## Discussion

This study represents a significant leap in the application of machine learning technology, specifically the random forest algorithm, for predicting postoperative complications in pediatric surgery. By leveraging this advanced algorithm, the authors have been able to dissect a complex array of clinical and laboratory data, enhancing the understanding of risk factors associated with ASBO following intestinal malrotation surgery in infants. This is in line with the recent trend of applying machine learning models to various aspects of surgical care, such as postoperative pain,^18^ wound infection,^19^ and mortality.^20^ However, to the best of our knowledge, this is the first study to use the random forest algorithm for predicting ASBO in pediatric patients, which is a challenging and clinically relevant problem.

The random forest model excels in handling multifaceted and non-linear data, typical challenges in clinical research.^21^ This enables accurate identification of key predictive variables, ranging from surgical duration to biochemical markers like pre-operative white blood cell count (pre-WBC) and post-operative albumin levels (post-ALB). Utilizing this method for risk prediction not only improves the understanding of postoperative courses but also aids in developing early intervention strategies. Previous studies have shown that early operative management of ASBO can be cost-effective and reduce the risk of recurrence and complications.^22^ However, the optimal timing and indications for surgery are still controversial and depend on various factors, such as the presence of strangulation, ischemia, or peritonitis.^13^ Therefore, having a reliable and robust predictive model can help surgeons to make informed decisions and tailor the treatment to individual patients. Additionally, the present study fills a significant gap in existing literature by focusing on the early prediction of ASBO in a previously underexplored demographic: infants undergoing intestinal malrotation surgery. The present research provides essential insights into postoperative risks for these young patients and offers a valuable predictive tool for clinicians to identify and manage ASBO early, which is crucial considering the vulnerability of this patient group and the potential for improved outcomes through early detection and intervention. The incidence of ASBO after laparotomy during infancy is reported to be between 1 and 12.6 %,^23^ and it can cause significant morbidity and mortality, especially in cases of recurrent volvulus or necrotizing enterocolitis.^6^ Therefore, it is imperative to identify the risk factors and preventive measures for ASBO in this population, as well as to monitor and treat them promptly if they occur.

The present research involved a retrospective analysis of clinical parameters from patients under three months old, focusing on key variables including pre-WBC, MV duration, surgery duration, and post-ALB. The model exhibited high diagnostic accuracy with an AUC of 0.960, indicating its potential for early identification of patients at risk for ASBO.

There are four variables with a feature importance greater than 10 %, pre-WBC, MV duration, surgery duration, and post-ALB. Elevated preoperative pre-WBC levels are indicative of a preoperative inflammatory state, potentially increasing the risk of postoperative complications such as ASBO. This underlines the importance of managing preoperative inflammation to mitigate such risks. Studies have consistently shown a strong correlation between preoperative leukocyte elevation or infection status and the occurrence of postoperative complications. For instance, Mahmood et al.^24^ found in cardiac surgery patients that elevated preoperative white blood cells were significantly associated with increased risks of 30-day mortality, wound complications, and other medical complications. Similarly, research focusing on risk factors for early postoperative ileus in elective colorectal surgery patients identified that variables like preoperative antibiotic use and the duration of antibiotic treatment were linked to a heightened risk of early postoperative ileus.^25^ These findings suggest that such factors might serve as indirect indicators of the effects of preoperative infection status on postoperative outcomes.

The present research reveals a direct correlation between the duration of surgery and the increased risk of ASBO. Prolonged surgical procedures often indicate a higher level of complexity or extensiveness, leading to more significant tissue damage and enhanced inflammatory responses. It has been established through studies that these inflammatory responses, resulting from tissue damage and the surgical process, can potentially trigger the formation of adhesions.^26^^,^^27^ Additionally, these postoperative adhesions are integrally associated with the body's healing mechanisms for damaged tissue, therefore, the development of adhesions is part of the body's natural response to surgical trauma, serving to heal and safeguard the affected area.^28^

The present study shows that prolonged postoperative MV use is associated with an increased incidence of ASBO. Research indicates that mechanical ventilation, particularly at high positive end-expiratory pressures, can diminish splanchnic perfusion. This reduction in blood flow, especially in the splanchnic area encompassing the gastrointestinal tract, significantly impacts gastrointestinal function.^29^ Consequently, prolonged MV after surgery is often indicative of a complex recovery process, heightening the risk of delayed bowel function and ASBO. Therefore, it is essential to limit the duration of MV to mitigate the risk of ASBO. Moreover, lower ALB levels post-surgery poses an additional risk, potentially worsening postoperative complications.^30^ A decline in ALB levels may reflect either compromised nutritional status or systemic inflammation, both detrimental to the healing process. Such decreases in ALB levels are frequently indicative of the body's stress response to surgery, which can amplify the likelihood of complications like ASBO by impairing immune function and delaying tissue repair.

However, this study's limitations include a modest sample size, its retrospective design, and data sourced from a single medical center. While the bootstrap method strengthens the present model's validation, further external validation in a broader, independent patient population is needed. The small sample size and single-center context limit the model's generalizability and the ability to establish causation. Future research should incorporate larger, multi-center, prospective studies to enhance data diversity, improve generalizability, and allow for more controlled data collection. Prospective studies are crucial for validating predictive models and establishing causal relationships. Integrating this model into various clinical settings will require adaptability across different patient populations. Future efforts should aim at refining the model for wider pediatric surgical contexts and maintaining transparency in its predictive processes for effective clinical decision-making.

In conclusion, this study introduces a promising machine learning-based method to predict ASBO in infants with intestinal malrotation post-surgery. The model's high accuracy and robust performance metrics underscore its potential in clinical decision-making, aiming to enhance patient management and improve outcomes in this vulnerable group. Future studies are necessary to validate and integrate this model into clinical workflows, thereby improving precision in pediatric surgical care.

## Funding

None.

## Authors’ contributions

Pengfei Chen and Zhenhua Guo contributed to the study concept and design and the data acquisition. Pengfei Chen and Haiyi Xiong performed statistical processing and analyses and drafted the initial manuscript. Haiyi Xiong, Jian Cao, Mengying Cui contributed statistical expertise. Pengfei Chen, Haiyi Xiong, Jinfeng Hou, Zhenhua Guo contributed to the drafting and critical revision of the manuscript.

## Conflicts of interest

The authors declare that the research was conducted in the absence of any commercial or financial relationships that could be construed as a potential conflict of interest.

## Appendix Supplementary materials

Image, application 1

## Footnotes

Supplementary material associated with this article can be found in the online version at doi:10.1016/j.jped.2024.11.011.
