# Supplementary material for: Predicting postoperative adhesive small bowel obstruction in infants under 3 months with intestinal malrotation: a random forest approach
Source: J Pediatr (Rio J). 2025 Jan 21;101(2):282–9. doi: 10.1016/j.jped.2024.11.011 (PMC11889664; doi:10.1016/j.jped.2024.11.011)
Supplement: Supplementary file 1 [file mmc1.docx]

**JPED-D-23-00528_ Supplementary Material**

**Supplementary Table S1** Performance metrics for ASBO prediction model with 95% confidence intervals

|  | Value | 95% CI |
| --- | --- | --- |
| AUC | 0.960 | 0.911 – 0.990 |
| Sensitivity | 0.805 | 0.600 – 0.950 |
| Specificity | 0.952 | 0.897 – 1.000 |
| Precision | 0.809 | 0.654 – 1.000 |
| F1 Score | 0.799 | 0.684 – 0.900 |

**Supplementary Figure S1** Feature selection using the boruta algorithm. The model highlights significant features in green and excluded features in red, yellow boxplots indicate tentative features, for which the algorithm does not provide a definitive recommendation on inclusion or exclusion. This study’s model includes only the variables highlighted in green.





**Supplementary Figure S2** Optimal hyperparameter tuning of random forest model: influence of “max_depth” and “n_estimators” on model accuracy. The horizontal and vertical axes represent the depth of the trees and the number of trees in the random forest model, respectively. The numbers in the heatmap indicate the model's accuracy for each parameter combination.


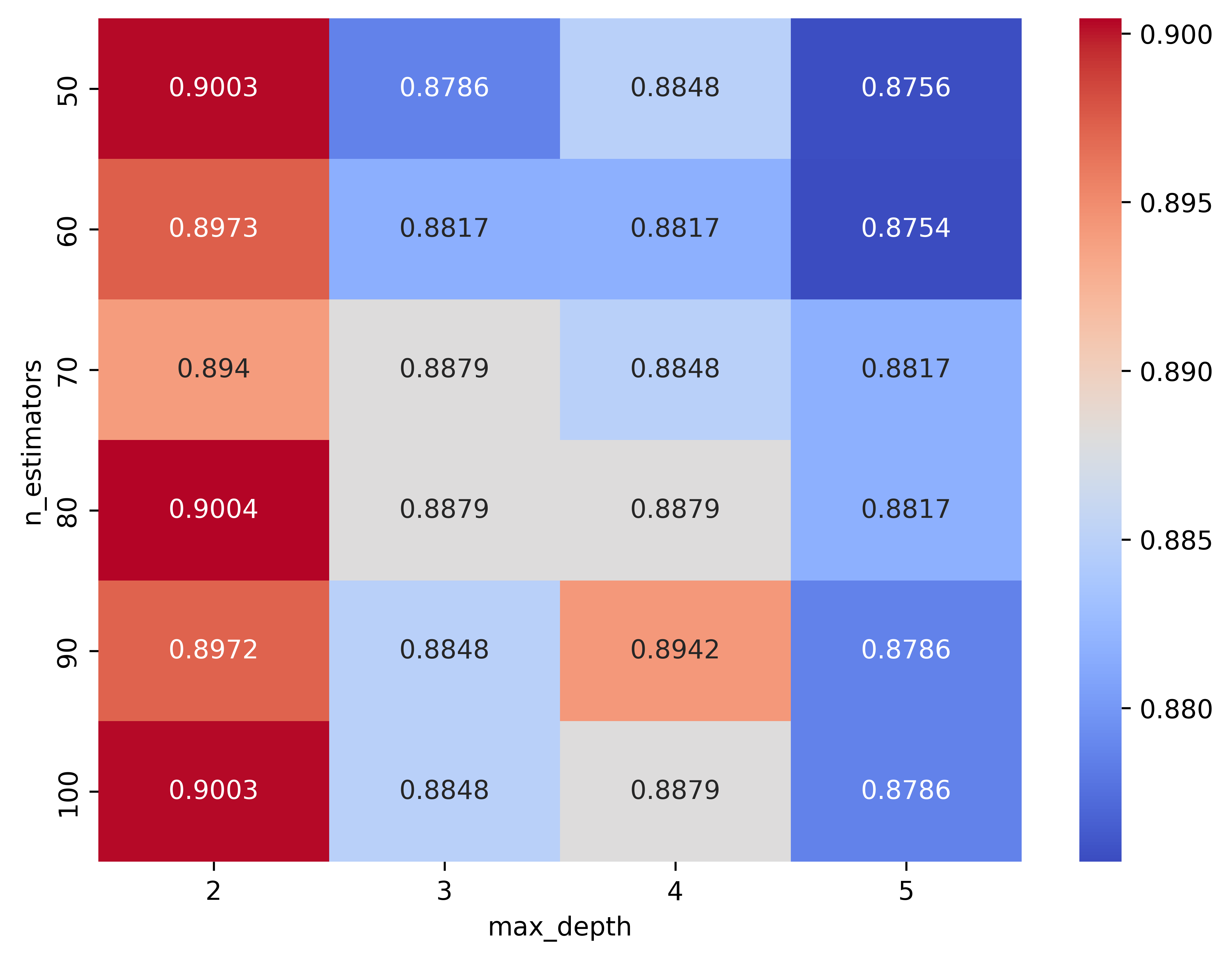


**Supplementary Figure S3** Partial dependence plots for predictive features in ASBO prediction.


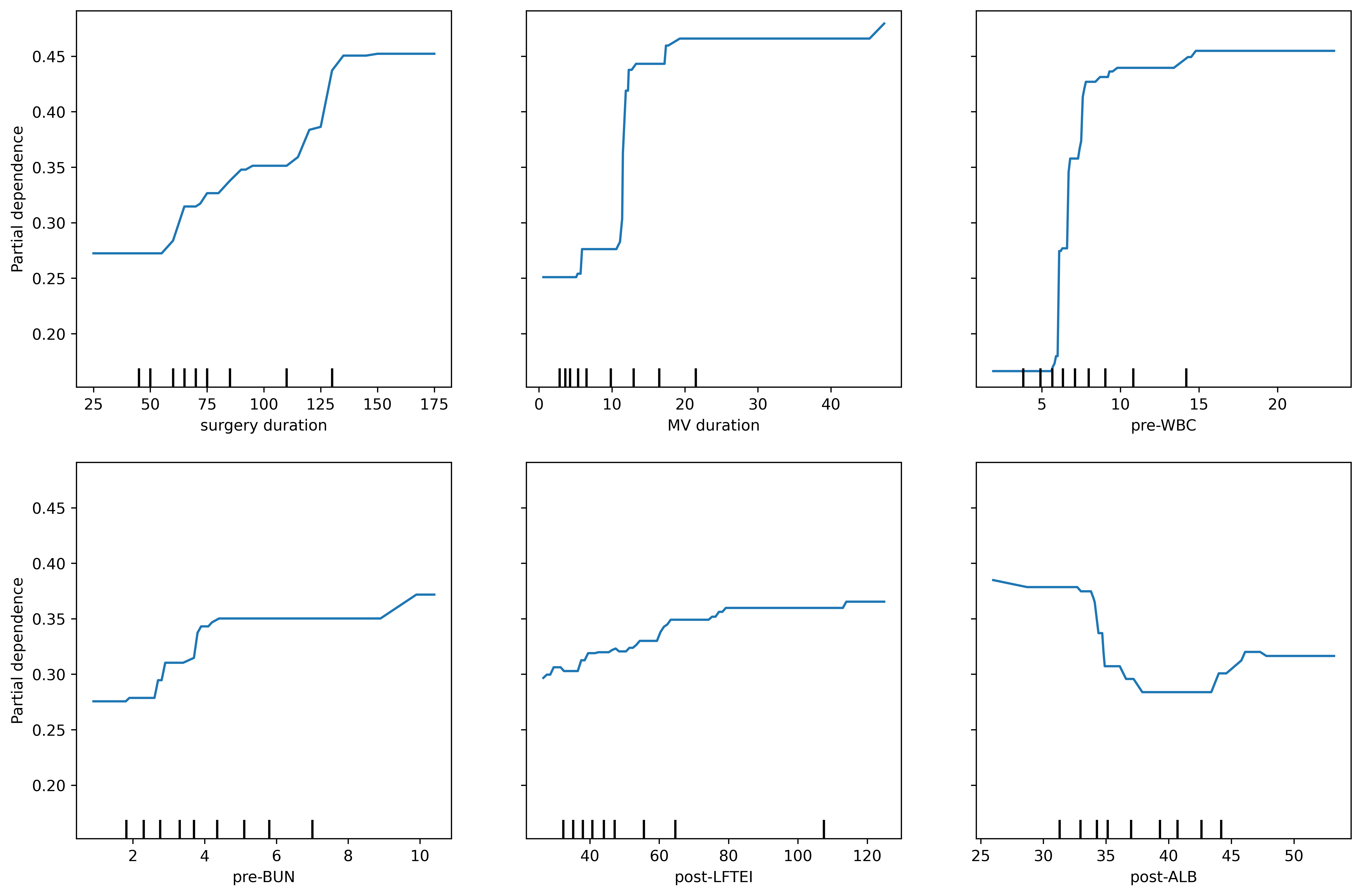


**Supplementary Figure S4** Visualization of six trees from the random forest composed of 80 trees.
